# Supplementary material for: The Relation of Surgical Procedures and Diagnosis Groups to Unplanned Readmission in Spinal Neurosurgery: A Retrospective Single Center Study
Source: Int J Environ Res Public Health. 2022 Apr 15;19(8):4795. doi: 10.3390/ijerph19084795 (PMC9028768; doi:10.3390/ijerph19084795)
Supplement: Supplementary file 1 [file ijerph-19-04795-s001.zip › ijerph-1666004-supplementary.pdf]

Supplementary Material

Table S1. Included diagnoses for the four diagnosis groups according to ICD-10-GM.

index diagnosis group	**ICD-10-GM**
**degenerative**	
cervical stenosis	M48.02
	M48.03
thoracic stenosis	M48.04
lumbar stenosis	M48.05
	M48.06
cervical herniation	M50.0
	M50.1
	M50.2
lumbar herniation	M51.0
	M51.1
	M51.2
listhesis	M43.1
**neoplasm**	
unknown tumor	D43.4
meningeoma	D32.1
benign neoplasm	D33.4
	D36.1
	D48.1
malign neoplasm	C72.0
	C72.1
plasmocytoma	C90.20
cyst	G96.1
	M70.8
**functional**	
chronic pain	G54.4
	K62.8
	M25.56
	M54.1
	M54.3-5
	M54.5

	M89.09
	M96.1
	R52.2
spasticity	S34.11
	G82
	G83
**other**	
discitis	M46.49
abscess	G06.1
	G06.2
epidural hematoma	G95.10
	S06.4
arteriovenous fistula	I77.0
arteriovenous malformation	Q28.88
hemangioma	D18.08
spina bifida	Q05
tethered cord	Q06.8
borreliosis	A68.1

ICD-10-GM, Classification of Diseases and Related Health Problems, 10th Revision, German Modification.

Table S2. Listing of operation and procedure codes according to the OPS coding system 2020.

Surgical access and procedures		OPS code
surgical access	subcutaneous	5-896
		5-039.d
		5-039.f/g/h/m/n/p/q
		5-059.c0/1
		5-059.d/g/h
	intraspinal extradural	5-030 ff
		5-031 ff
		5-032 ff
		5-083.1
		5-832
		5-033.0
	material implantation	5-836
		5-83b.5
		5-83b.7
		5-039.d
		5-837
		5-039.e/f/g/h/m/n/p/q
		5-059.c0/1
		5-059.d/g/h
		5-038 ff
		5-039.32-39
		5-039.8
		5-039.a/b/c without 5-039.a8/9
		5-039.c6/7
		5-039.a8/9
		5-039.c6/7
		5-039.j/k
	intraspinal intradural	5-039.8
		5-039.a6/7
		5-039.c4/5
		5-039.g
		5-034.0/1/2
	intraspinal intradural intramedullary	5-035.0

		5-035.1
		5-034.3/4/5
surgical procedure	sequestrectomy and nucleotomy	5-831
		5-830.1
	spinal decompression	5-832
		5-033.0
	intervertebral cage fusion	5-83b.7
	spondylodesis	5-836
		5-83b.5
	spinal cord stimulation	5-039.32-39
		5-039.8
		5-039.a/b/c without 5-039.a8/9
		5-039.c6/7
	pulse generator implantation	5-039.d
		5-039.f/g/h/m/n/p/q
		5-059.c0/1
		5-059.d/g/h
	resection of intraspinal tumor, except for neurinoma	5-035.0
		5-035.1
		5-035.2
		5-035.3
		5-035.4
		5-035.5
	resection of intraspinal neurinoma	5-035.6
		5-035.7
		5-041
	wound débridement	5-896
	medication pump implantation	5-038 ff
	peripheral nerve stimulation	5-059.8
		5-059.9
		5-059.a
		5-059.c
	resection of intraspinal empyema	5-034.2
		5-033.3
	resection of intraspinal hematoma	5-033.2

		5-034.1
		5-034.4
	corporectomy	5-837
	dorsal root ganglion stimulation	5-039.a8/9
		5-039.c6/7
		5-039.j/k
	resection of arteriovenous malformation	5-399.1
	lumbar puncture	1-204.2
	biopsy	1-502.4
	cancel/abort procedure	5-995
	resection of spinal abscess	5-034.5

OPS code, operation and procedure code.

Table S3. Predictors for unplanned readmission. Univariate logistic regression for demographic data und hospital characteristics.

Univariate regression	OR (95% CI)	p-value
**total cohort**		
age, >48 years	2.39 (1.063-5.363)	0.035
PCCL, >7	2.35 (1.332-4.163)	0.003
discharge at own discretion	4.56 (0.523-39.772)	0.170
surgery, ≥2 interventions	2.94 (1.093-7.891)	0.033
night shift surgery [a]	49.61 (4.396-559.85)	0.002
comorbidity [b]	2.03 (1.147-3.575)	0.015
previous organ transplantation	11.43 (1.019-128.195)	0.048
index diagnosis groups		
lumbar herniation	0.51 (0.246-1.064)	0.073
cervical stenosis	3.81 (0.520-27.96)	0.188
lumbar stenosis	2.05 (1.150-3.649)	0.015

abscess	22.88 (1.410-371.179)	0.028
chronic pain	1.74 (0.893-3.405)	0.103
**surgical group**		
age, >50 years	3.30 (1.275-8.53)	0.014
LOS, >6 days	2.67 (1.385-5.132)	0.003
comorbidity[b]	2.40 (1.241-4.629)	0.009
surgeries, ≥2 interventions	2.94 (1.093-7.891)	0.033
diabetes mellitus	2.83 (0.674-11.919)	0.155
previous organ transplantation	12.10 (1.072-136.405)	0.044
surgical access		
subcutaneous	3.22 (0.710-14.624)	0.129
intraspinal extradural	1.58 (0.826-3.034)	0.167
surgical procedure		
Wound débridement	3.74 (0.814-17.212)	0.090
sequestrectomy and nucleotomy	2.40 (1.042-5.501)	0.040
spondylodesis	2.16 (0.874-5.340)	0.095
resection of intraspinal neurinoma	3.47 (0.760-15.854)	0.108
Generator implantation	2.43 (0.824-7.182)	0.107
dorsal root ganglion stimulation	9.82 (1.843-52.352)	0.007
index diagnosis group		
lumbar herniation	2.41 (0.995-5.824)	0.051
lumbar stenosis	2.29 (1.183-4.44)	0.014
abscess	24.22 (1.486-394.754)	0.025
benign tumor	4.82 (0.549-42.31)	0.156

chronic pain	1.74 (0.804-3.750)	0.160
**non-surgical group**		
gender, female	3.55 (0.937-13.47)	0.062
PCCL, >1	4.86 (1.193-347.539)	0.016
discharge		
at own discretion	20.36 (1.193-347.539)	0.037
external hospital	20.36 (1.193-347.539)	0.037
home	14.80 (2.218-98.760)	0.005

CI, 95% confidence interval; *LOS,* length of stay; *OR,* odds ratio, *PCCL,* patient clinical complexity level. [a] night shift: 7 p.m. until before 7 a.m. [b] comorbidity: defined as five or more side diagnoses
